# Topic Modeling of Social Media Discourse of Autism Support Groups

**DOI:** 10.3390/bs16020280

**Published:** 2026-02-15

**Authors:** Yu Deng, Lei Yang, Juanjuan Chen

**Affiliations:** 1 Center for Linguistic, Literary & Cultural Studies, Sichuan International Studies University, Chongqing 400031, China; 2 College of Language Intelligence, Sichuan International Studies University, Chongqing 400031, China; 3 Institute of Educational Planning and Assessment, Sichuan International Studies University, Chongqing 400031, China

**Keywords:** autism support groups, social media, topic modeling, discourse analysis

## Abstract

Social media platforms serve as critical channels for autism support groups to communicate and seek assistance. This study employed Latent Dirichlet Allocation (LDA) topic modeling to analyze discourse patterns within the Autism Bar on Baidu Tieba, a major Chinese social media. A dataset of 14,151 posts was collected through web crawling, with 12,667 posts retained after preprocessing. The analysis revealed two key findings: (1) The discourse among autism support communities on Baidu Tieba focuses on four central themes: intervention and therapy, early educational journey, early symptom detection and family interaction, and access to educational resources and community support. (2) Sociocultural factors exert a significant influence on autism-related discourse, particularly in shaping societal attitudes toward individuals with autism and the formation of support networks. Traditional Chinese cultural values, such as collectivism and familial centrality, impact the behavioral patterns and decision-making processes of families with autistic children. This study has demonstrated the unique needs and challenges faced by the autism support community, while also informing strategies to promote social media platforms as spaces for support and information exchange. The findings have practical implications for designing targeted interventions and support mechanisms for individuals with autism and their families.

## 1. Introduction

Autism spectrum disorder (ASD) is a neurodevelopmental condition characterized by challenges in social interaction, communication, and restricted, repetitive behaviors (American Psychiatric Association, 2013). The prevalence of ASD has been increasing globally, with recent estimates suggesting that approximately 1 in 31 children are diagnosed with ASD in the United States (Shaw, 2025). This rise can be attributed to better awareness, broader diagnostic criteria, and improved reporting systems (Lyall et al., 2017). In China, ASD awareness and diagnostic rates are also increasing, consistent with global trends (Sun et al., 2013; W. Zhou & Guan, 2024). However, societal understanding and support systems for ASD in China differ markedly from Western contexts due to cultural factors and variations in healthcare infrastructure (X. Li et al., 2024; Q. Zhou et al., 2023).

Families of children with ASD are likely to experience heightened levels of stress and emotional burden. Studies have shown that caregivers of children with ASD face more significant challenges than parents of neurotypical children, including higher levels of stress, anxiety, and depression (M. E. Dunn et al., 2001; Napitupulu & Kurniawan, 2023). In the Chinese context, these challenges are compounded by social stigma and a lack of support systems (X. Li et al., 2024; Tang & Bie, 2016; W. Zhou & Guan, 2024).

The advent of social media has offered a new platform for social support and information sharing for families affected by ASD. ASD online support groups are virtual communities on social media designed for individuals with autism, their families, caregivers, and professionals. Within these spaces, members engage in communication, share experiences, and provide mutual support (Corti et al., 2022). Online communities on social media can provide critical informational and emotional support, and can thus reduce feelings of isolation for families dealing with ASD (Gupta & Singhal, 2005; Zhao et al., 2019). Online support groups on platforms such as Twitter, Reddit, Baidu Tieba, and Weibo offer an online platform for sharing lived experiences, seeking advice, and finding emotional support.

The existing literature on social media discourse within autism support groups reveals a dynamic and multifaceted domain where autistic individuals and caregivers seek information, community, and identity validation (Duffin et al., 2025; Larnyo et al., 2024; Lorence, 2007; Nguyen et al., 2014; Skafle et al., 2024; Zhao et al., 2019). For instance, Lorence’s (2007) grounded theory analysis of online discussions regarding Asperger’s syndrome framed social media forums as “virtual support groups”. In online spaces, the peer-to-peer exchange of information was found to inform medical decision-making, thus constituting a form of “cybertherapy”. Building on a computational comparative approach, Nguyen et al. (2014) demonstrated that autism-focused online communities exhibit distinct linguistic patterns, topics, and a lower emotional valence indicative of poorer mood compared to control groups, thereby underscoring the diagnostic and supportive potential of social media data. Zhao et al. (2019) conducted a thematic analysis of Facebook data from autism support groups to identify their core discursive representation, including the exchange of practical advice, emotional support, and personal narratives. The findings solidified the concept of social media platforms as vital channels for peer-led knowledge and communal “voice”. Most recently, Larnyo et al. (2024) investigated Reddit discussions to reveal the health information needs of the ASD community in the post-COVID-19 era. Their analysis identified salient themes such as diagnostic challenges and stigma, paradoxically uncovering a predominance of positive sentiment alongside a pronounced need for more reliable digital resources. This tension between support and informational inadequacy is echoed by Skafle et al. (2024), who highlighted the dual role of social media: while it provides vital identity-related information absent from official channels, it can also foster alienation through hostile in-group discourse. Complementing these insights into user experience, Duffin et al. (2025) provided a novel model of the information journeys of autistic adults, showing how online groups are structurally integral to seeking and sharing autism-related information, thereby addressing a critical gap in understanding information behaviors of autistic individuals.

In the context of China, previous studies exploring ASD discourse within Chinese digital spaces demonstrated several culturally mediated influences on ASD-related communication and support-seeking, including the evolving structure of internet discourse, the role of traditional media in shaping public perceptions, prevalent parental knowledge gaps, and the impact of cultural values on family stress and coping mechanisms (Huang & Zhou, 2016; H. Li, 2019; Ng et al., 2021; Su et al., 2021; Wang et al., 2022). Most recent research on autism within Chinese social media has concentrated on topics and public attitudes toward autism (X. Li et al., 2024; Q. Zhou et al., 2023). Drawing on Zhihu data, Q. Zhou et al. (2023) identified discussion topics among autistic individuals and caregivers, uncovering critical issues such as the prevalence of negative emotions, public stereotyping, and the significant financial and psychological stress borne by families. Complementing this, X. Li et al. (2024) examined a decade of Weibo posts, revealing an overall positive and improving public attitude toward the autism community, particularly in affective and behavioral dimensions, yet simultaneously confirming an underdeveloped cognitive understanding that aligns with the stereotypes and prejudices noted by Q. Zhou et al. (2023). While these studies highlight the necessity of culturally sensitive interventions that integrate traditional values, address information deficits, and leverage digital platforms, they have underestimated the specific influence of cultural and social contexts—particularly the Chinese context—on the needs and behaviors of online ASD support groups (Zhang et al., 2024). This oversight represents a critical gap in understanding how cultural specifics, such as collectivism and face-saving behaviors, directly shape the experiences, interactions, and perceived support within these digital communities (X. Li et al., 2024; Q. Zhou et al., 2023).

Regarding methodologies for analyzing discussions within ASD online support groups, a predominant reliance on self-reported measures is limited in its ability to capture the nuanced dynamics of online interactions (Y. Liu, 2014). Instead, a direct examination of the content of social media discourse—such as prevalent themes, linguistic patterns, and interaction structures—can yield more robust insights into the lived experiences, needs, and challenges faced by individuals and families affected by ASD. Along this line of discourse analysis of online support groups, text mining methods such as topic modeling, informed by data obtained through web crawling, function as an effective big data approach to the portrayal of these dynamics (Bellon-Harn et al., 2022; Corti et al., 2022; Nguyen et al., 2014). For instance, Corti et al. (2022) examined discourse on Twitter, analyzing prevalent topics and user attitudes concerning ASD and the indirect impact of COVID-19. Employing Non-Negative Matrix Factorization (NMF) for topic modeling, they demonstrated that Twitter facilitates substantial discourse on ASD, particularly emphasizing themes related to family dynamics, community support, and therapeutic interventions.

Taken together, the literature illustrates that ASD online support communities are critical spaces for information exchange and social connection, forming complex socio-informational ecosystems for virtual communication. It is noted that previous research somehow underestimated social media’s role in disseminating knowledge about ASD. This gap indicates a need to examine social media discourse among individuals connected to ASD, highlighting the platform’s potential in promoting awareness and understanding of supporting those with ASD. This study aims to employ Latent Dirichlet Allocation (LDA) topic modeling to explore the discourse of Chinese autism support groups on the social media Baidu Tieba, a major Chinese online forum platform operated by the Chinese technology giant Baidu and open to people around the world who use the Chinese language. Baidu Tieba is structured around topic-based communities (“bars”) where users discuss shared interests. Primarily serving Chinese users, it requires registration with a phone number but allows pseudonymous interaction. While accessible via web browsers and mobile apps, Baidu Tieba competes with platforms such as Douban, Zhihu, and Weibo in facilitating niche discussions. Its design emphasizes open, search-driven communities, functioning as a hybrid of traditional forums and modern social media. In the present study, LDA was employed to identify the underlying themes and topics present in the preprocessed data collected from Baidu Tieba “Autism Bar”. Fairclough’s (1992) three-dimensional model of critical discourse analysis was utilized to interpret the social media discourse of Chinese ASD support groups. This method provides a way to gain a deeper understanding of the topics prevalent in ASD online communities, as well as the social practices that shape these interactions. This study contributes positively to the awareness and understanding of autism within the online community. It attempted to answer the following questions:

(1) What are the predominant topics of engagement in the autism groups on Baidu Tieba, and how do they reflect the ASD community’s concerns and needs?

(2) How do cultural and social contexts within China influence online ASD support groups’ dynamics on Baidu Tieba?

## 2. Materials and Methods

### 2.1. Study Design

**Figure 1:**
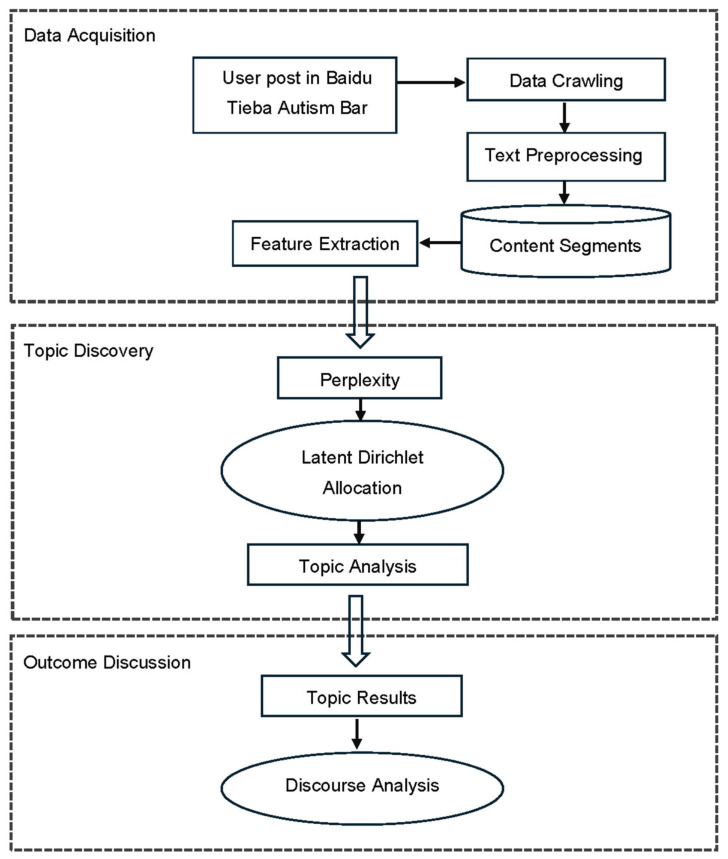
The overall study design.

The overall framework is illustrated in Figure 1. This study was designed to investigate the dynamics within ASD support groups on Baidu Tieba, concentrating on the Autism Bar (自闭症吧). We conducted an exhaustive crawling of all discussions in the “Autism Bar” on Baidu Tieba spanning the decade from 2013 to 2023, thereby ensuring a robust dataset for analysis. Python (version 3.9) scripts were created utilizing the Natural Language Toolkit (NLTK), a method informed by its effectiveness in processing linguistic data (Bird et al., 2009). These scripts were systematically interfaced with Baidu Tieba’s API to retrieve all available posts from the Autism Bar, ensuring a complete capture of the community’s discourse over an undefined period until the data were fully harvested (Loper & Bird, 2002).

Data preprocessing involved refining the raw textual data by removing HTML tags, stopwords from NLTK’s repository, punctuation, white spaces, and URLs. This essential cleaning process improved the data quality for text analysis in natural language processing (Haddi et al., 2013). Latent Dirichlet Allocation (LDA) topic modeling was adopted, a method lauded for its utility in uncovering underlying thematic structures within text corpuses (Blei et al., 2003). The number of topics and their clarity were optimized by evaluating the model’s perplexity, adhering to established model-fitting criteria (Blei et al., 2003; J. Liu et al., 2020). The LDA models were assessed and visualized using pyLDAvis, an interactive tool that facilitated the exploration and interpretation of the topics by providing a user-friendly representation of the model’s topics and terms (Sievert & Shirley, 2014; Yuan et al., 2024). This visual evaluation was instrumental in understanding the distribution and distinction between topics (Chuang et al., 2012).

The final interpretive stage utilized Fairclough’s (1992) three-dimensional model, which conceptualizes discourse as an integrated structure comprising text, discursive practice, and social practice. This model involves: (1) the linguistic description of the text; (2) the interpretation of the relationship between discursive processes—both productive and interpretive—and the text; and (3) the explanation of how these discursive processes relate to broader social processes (Fairclough, 1995). Employing Fairclough’s (1992, 1995) three-dimensional model provided a comprehensive understanding of both the linguistic and sociocultural layers of the discourse within the ASD support groups on Baidu Tieba.

### 2.2. Data Collection

#### 2.2.1. Selection of Social Media Platform

This study selected Baidu Tieba as the data platform, focusing on the Autism Bar (自闭症吧). Baidu Tieba, operated by Baidu Inc. (Beijing, China), is a leading Chinese online forum that organizes anonymous user discussions into topic-specific sub-communities known as “bars”(J. Liu et al., 2020; Yuan et al., 2024). As of December 2021, the platform reported 1.5 billion registered users, with 45 million monthly active users and over 23 million distinct communities. This platform functions as a vital resource for individuals accessing ASD-related information and support, while also fostering both informal and substantive discourse on topics of mutual interest.

The Autism Bar, which was established in 2006, serves as a dedicated online community for individuals affected by or interested in ASD. At the time of data collection (November 2023), it had approximately 84,000 followers, generating substantial discourse due to high user engagement. Our dataset comprised primarily original posts in which members exchanged information, personal experiences, and mutual support. This active and focused environment on Baidu Tieba provided rich textual data for analyzing ASD-related discourse.

#### 2.2.2. Data Crawling

An exhaustive crawling of the Autism Bar was conducted to obtain a comprehensive dataset. The crawling, executed through the Baidu Tieba API, facilitated a systematic extraction of posts and their associated metadata (J. Liu et al., 2020). Following ethical guidelines of Sichuan International Studies University and platform policies of Baidu Tieba, we employed web crawling to collect textual data while ensuring user privacy by excluding all personally identifiable information. The crawling was conducted in such a manner as to not disrupt the forum’s normal operations or infringe on user privacy (J. Liu et al., 2020; Marwick & Lewis, 2017). The data collection process was designed to be inclusive of both historical and current discussions, capturing a wide spectrum of user interactions and topics. The specified timeframe for data acquisition was from July 2013 to October 2023, allowing for a varied and rich dataset that reflected the evolving nature of online discourse (Marwick & Lewis, 2017).

The crawling process was facilitated through Python scripts and bifurcated into two distinct stages. In the initial phase, the script navigated through the Autism Bar’s sections, collecting basic information such as post links (“链接”) and titles (“标题”). This information was systematically recorded in a CSV file named “百度贴吧帖子.csv”. This preliminary data compilation provided a foundational index of forum posts, which was pivotal for the subsequent, more granular data retrieval process. The second stage of the crawling harnessed the list of post links generated during the first phase. The script then accessed each link to collect detailed data from individual posts. The extracted information included the post’s content, the timestamp of posting, the poster’s IP address location, and the username. To maintain user anonymity, only the general location data derived from the IP address was collected rather than any precise geolocation information. The extracted details were organized into a CSV file with dedicated columns for “链接” (link), “标题” (title), “内容” (content), “发文时间” (posting time), “ip归属地” (IP address location), and “用户名” (username). Initially, the data crawling process yielded a total of 14,151 posts. Based on the available IP location data, the user demographics of the platform covered all 31 provincial-level administrative regions of mainland China, including municipalities and autonomous regions. The user base was prominently represented in populous and economically active provinces such as Guangdong, Hebei, Jiangsu, Zhejiang, Shandong, Henan, Beijing, Shanghai, Sichuan, Hunan, Hubei, Fujian, Jiangxi, Anhui, Liaoning, Shaanxi, Shanxi, Guangxi, Yunnan, Chongqing, Heilongjiang, Jilin, Tianjin, Guizhou, Gansu, Inner Mongolia, Xinjiang, Hainan, Ningxia, Qinghai, and Tibet. This confirms the platform’s extensive geographic reach and diverse regional user participation.

### 2.3. Data Preprocessing

#### 2.3.1. Text Cleaning

The preprocessing of the dataset was a fundamental step to ensure the cleanliness and analytical relevance of the text obtained from the Autism Bar on Baidu Tieba. The raw data typically included noises such as URLs, special characters, and non-Chinese text, and thus a thorough cleaning process was necessary.

The initial phase of preprocessing involved the application of text-cleaning techniques to remove extraneous and non-contributory elements from the dataset, involving the correction of spelling errors, removal of formatting errors, and handling of missing values (Bird et al., 2009). The cleaning process utilized regular expressions to identify and remove hyperlinks, special characters, emoticons, and alphanumeric noise that could interfere with the text analysis process.

For instance, two raw posts initially present as follows:

(1) “来自：http://example.com 这个方法👍很有效！😊” (translated: “From: http://example.com This method👍 is very effective!”).

(2) “每天进步一点点，从量变到质变✊#特殊儿童##儿童康复训练#【图片】【图片】【图片】” (translated: “Progress a little every day, from quantitative change to qualitative change✊ #SpecialChildren# #ChildrenRehabilitationTraining#[Image][Image][Image]”).

After the cleaning process, example (1) was transformed into “这个方法很有效” (“This method is very effective”), with URLs, emoticons, and special characters removed, ready for further analysis. The cleaning process of example (2) removed non-textual elements such as emoticons (“✊”), hashtags (“#特殊儿童#”, “#儿童康复训练#”), and repeated non-informative placeholders for images (“【图片】”). The post was refined to “每天进步一点点，从量变到质变” (“Progress a little every day, from quantitative change to qualitative change”). The cleaned version removed distractions, focusing on the meaningful content for analysis of topics.

Furthermore, a content length filter was implemented. Posts encompassing four or fewer characters, such as “他人分享” (“shared by others”), lacked sufficient content for meaningful analysis. These posts were considered non-valuable for the lack of contextual information or depth for topic modeling, and were removed from the dataset. The text cleaning attempted to improve the accuracy and reliability of the subsequent topic modeling, focusing on more substantial and informative content from the discussions within the Autism Bar.

#### 2.3.2. Removal of Stopwords

The dataset was refined through the removal of stopwords, a standard preprocessing step in natural language processing (NLP). Stopwords (e.g., “and”, “the” in English; “和”, “是”, “呢” in Chinese) that contribute minimal semantic meaning were filtered from Chinese text data collected from the Autism Bar. Using a predefined Chinese stopword list (Che et al., 2021), conjunctions, articles, and prepositions were excluded to retain lexically meaningful content. This process enhanced the dataset’s analytical utility by isolating words and phrases critical for topic modeling, thereby improving the extraction of substantive discourse patterns from the forum. An example is given below:

(3) “但是，这个方法，尽管简单，但效果显著。” (translated: “However, this method, although simple, has a significant effect.”).

After stopwords such as “但是” (“however”) and “尽管” (“although”) were removed, the refined sentence focused on the essential content: “这个方法简单，效果显著。” (“This method is simple, has a significant effect.”).

#### 2.3.3. Tokenization

Tokenization is the process of breaking down the text into individual words or tokens (Jing & McKeown, 2000). As Chinese words are not separated by spaces, tokenization is crucial for delineating individual words or tokens within the text (Jing & McKeown, 2000). The study utilized the Jieba split-word tool designed for Chinese text, which segmented the cleaned dataset into meaningful units or tokens. An example is given below:

(4) “自闭症儿童需要更多关爱” (translated: “Children with autism need more love”).

Based on the tokenization tool, the sentence is segmented into tokens: “自闭症”, “儿童”, “需要”, “更多”, “关爱” (“autism”, “children”, “need”, “more”, “love”). Tokenization converted raw text into analyzable units, enabling precise identification of key terms for topic modeling.

After rigorous preprocessing steps, the dataset was refined to 12,667 posts for discourse analysis.

### 2.4. LDA Topic Modeling

#### 2.4.1. About LDA

Latent Dirichlet Allocation (LDA), a probabilistic topic modeling approach developed by Blei et al. (2003), identifies latent thematic structures in document collections by treating each document as a mixture of topics and each word as attributable to specific topics. As a foundational technique in machine learning and natural language processing, LDA employs Bayesian inference to model documents as random topic mixtures, where topics are characterized by distinct word distributions (Griffiths & Steyvers, 2004; Hoffman et al., 2010; Yuan et al., 2024; Zhao et al., 2019). The model requires predefined parameters (topic count, words per topic, and topics per document) and iteratively optimizes them to reveal underlying semantic patterns through word–document co-occurrence analysis.

#### 2.4.2. Model Training

To perform LDA topic modeling, the preprocessed text data was used to train the LDA model. The model learns the distribution of topics across the entire corpus of text documents. LDA assigns each document a mixture of topics and each topic a distribution of words. In the present study, the LDA model was implemented in Python with a gensim package using the Gibbs sampling inference method. The hyperparameter α was set to 0.1, while β equaled 0.01. The number of iterations was set as 500. The hyperparameters α and β were set according to values empirically validated as effective across diverse text corpora (Griffiths & Steyvers, 2004).

#### 2.4.3. LDA Model Evaluation

One crucial aspect of LDA modeling is the determination of the optimal number of topics, the K parameter, which determines the number of topics the LDA model is going to retrieve during the training process. In this study, interactive visualization methods (pyLDAvis package version 3.3.1 imported in Python 3.9) were employed to select the most appropriate number of topics that effectively capture the structure of the data (Griffiths & Steyvers, 2004; Yuan et al., 2024). Using the methods in previous studies (Jelodar et al., 2019; J. Liu et al., 2020; Zhao et al., 2019), the number of LDA topics was tuned until it reached a set of non-overlapping clusters that had sufficient distance between each other. The parameter of K was given for a series of descending numbers to train the model until the circles representing the topics became separated without any significant overlapping. After the ideal LDA models were produced, the revealed topics in each group were named manually according to the top terms in the topic–term distribution.

#### 2.4.4. Topic Labeling

Once the LDA model was trained and the number of topics was determined, the topics were interpreted and labeled based on the most representative words within each topic. This step involves human judgment and domain expertise to assign meaningful labels to the discovered topics. For a complete analysis, each of the topics was labeled with descriptive titles that encapsulate their thematic content. These labels serve to summarize and communicate the essence of the conversations occurring within each topic, offering a quick and intuitive understanding of the structure and content of the discussions within the ASD support groups.

## 3. Results

LDA topic modeling of Baidu Tieba’s Autism Bar dataset uncovered four distinct topics, each capturing key themes in the community’s discussions about ASD. The topics, named based on their most representative terms (Table 1), reflect the primary concerns and interests of forum members.

Topic 1, labeled as “autism intervention and therapy”, encompasses discussions on treatment options, rehabilitation techniques, and developmental interventions for children with autism. This theme highlights the community’s engagement with the practical aspects of managing autism, including parental involvement in therapies and the exploration of new and effective treatments.

Topic 2, termed “early childhood autism and education”, centers on the role of parents and educators in supporting children with autism. It concerns the early educational journey, integrating children into social environments such as kindergartens, and the challenges and hopes parents express for their children’s development.

Topic 3, referred to as “early childhood symptoms and family interaction”, delves into the familial aspects of raising children with autism. It covers topics related to early detection, the significance of parental observations in identifying early signs, and the pursuit of early interventions to support children’s growth.

Topic 4, named “educational resources and community support”, is characterized by discussions on the resources available for autism support, such as special education programs, rehabilitation centers, and community support mechanisms. It also includes conversations about navigating the healthcare system and sharing educational materials and experiences within the community.

### 3.1. Autism Intervention and Therapy

**Figure 2:**
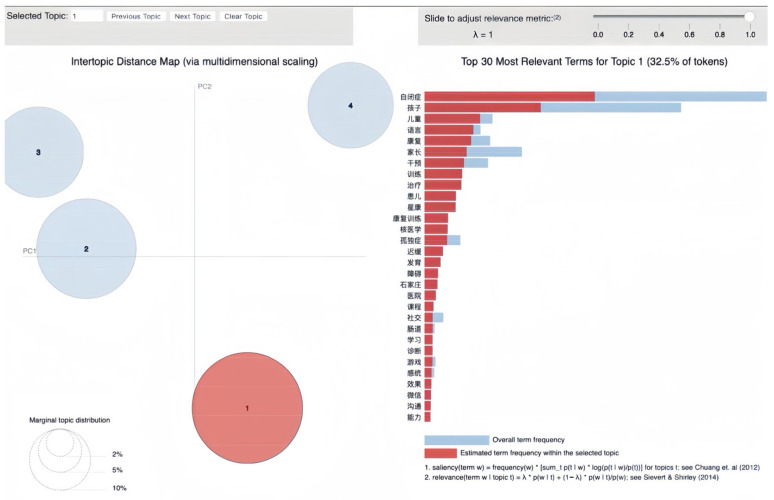
Visualization of Topic 1: autism intervention and therapy.

Topic 1 comprises 32.5% of the tokens in the corpus. It presents the community’s focus on treatment, development, and support, which are critical concerns for individuals and families dealing with ASD. The bar chart in Figure 2 reveals a nuanced array of terms that characterize this topic, providing insights into the thematic focus of the discourse among the ASD support groups.

Topic 1 stands out with its concentrated emphasis on “autism intervention and therapy.” The visualization, with red bars indicating the highest relevance, highlights terms like “自闭症” (autism spectrum disorder), “康复” (rehabilitation), and “训练” (training), drawing attention to the community’s engagement with therapeutic approaches. These terms tend to dominate the discourse due to their direct relation to the methods and efficacy of interventions for ASD.

The blue bars, perhaps indicative of slightly lower prominence within the topic yet still significant, showcase terms such as “语言” (language), “社交” (social interaction), and “感统” (sensory integration), pointing to the broader areas of concerns within the intervention strategies. These dimensions are critical for the holistic development of children with ASD, suggesting that the discussions are not just about treatments but also about improving key life skills.

The presence of terms such as “家长” (parents) and “患儿” (pediatric patients) shows active conversations around the vital role of the family in the intervention process. Keywords such as “石家庄” (Shijiazhuang) and “医院” (hospital) demonstrate location-specific discussions, possibly providing community members with relevant and accessible information regarding where they can seek interventions.

A representative post from the data reflects the community’s concerns about intervention and therapy:

(5) “有家长问：早期干预，能治愈彻底根治自闭症吗？早发现、早诊断、早干预的意义…0–6岁是儿童发展的关键期，但不等于错过了这一有利时机，干预就没有作用了…” (translated: “A parent asked: Can early intervention completely cure autism? The importance of early detection, early diagnosis, and early intervention… Ages 0–6 are a critical period for child development, but missing this opportune time does not mean that intervention is ineffective thereafter…”).

This post illustrates a central concern in Topic 1’s discourse: the potential and limits of early intervention and the crucial window of early childhood in ASD treatment and management.

It is noteworthy that the terms “石家庄” (Shijiazhuang) and “星康” (Xingkang) occur with notable frequency at the top of the list for Topic 1. “Xingkang” refers to a well-known facility specializing in autism intervention in “Shijiazhuang” that has made a considerable number of posts in the Autism Bar. The presence of these terms indicates that discussions may be influenced by the contributions from specific organizations or facilities that are active in the community. Autism rehabilitation services in China are predominantly concentrated in major wealthy urban centers, while remote and rural areas face severe shortages, thus forcing families to seek care across regions. Shijiazhuang Xingkang Autism School is conveniently located just one hour from the capital Beijing by high-speed rail, providing nationwide access to autism rehabilitation services. With more affordable pricing than Beijing-based options, it offers a practical choice for long-term intervention. This is especially advantageous for families who receive a diagnosis in Beijing’s major hospitals but seek sustained support, enabling seamless coordination between medical care in Beijing and professional rehabilitation in Shijiazhuang city, Hebei province.

The Autism Bar also features numerous active users representing facilities and education centers. Their posts shed light on therapy and intervention practices while potentially biasing discussions toward institutional perspectives. This highlights the need to account for post sources when interpreting LDA results, as they may reflect these entities’ specialized interests within the broader autism conversation.

### 3.2. Early Childhood Autism and Education

**Figure 3:**
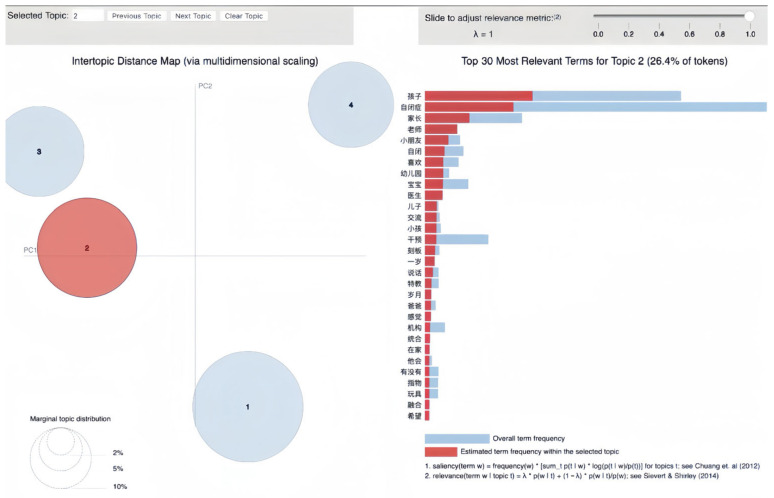
Visualization of Topic 2: early childhood autism and education.

The second major topic, represented by the prominent red circle in the intertopic distance map in Figure 3, accounts for 26.4% of tokens and constitutes another key focus in ASD support group discussions. The size of the red circle in the intertopic distance map shows the topic’s prevalence within the dataset.

Topic 2 illustrates conversations around “early childhood autism and education”. The dominant red bars, which indicate the most prominent terms, highlight “孩子” (children), “自闭症” (ASD), and “家长” (parents). This pattern suggests a strong discursive emphasis on how ASD affects early childhood and the central role parents play in this context.

The mention of “老师” (teacher) and “幼儿园” (kindergarten) among the top terms reveals dialog concerning educational settings and professionals’ roles in the development and care of children with ASD. These terms likely correspond to shared experiences, strategies, and advice on how to navigate educational systems, emphasizing the intersection of healthcare and education in early autism intervention.

Terms such as “交流” (communication), “说话” (speaking), and “刻板” (stereotypical behaviors) indicate that members of the forum are not only seeking information but are also exchanging knowledge about the symptoms and behaviors associated with early childhood autism. The repetitive occurrence of “干预” (intervention) underscores a proactive approach within the community towards early intervention, which is considered to be critical for the long-term rehabilitation for children with ASD.

The presence of words such as “儿子” (son), “爸爸” (father), and “在家” (at home) reflects a personal aspect of the discussions, emphasizing family experiences and the role of the home environment in supporting children with autism. The term “特教” (special education) further illustrates the focus on specialized educational approaches designed for the unique needs of these children.

The visualization indicates that “玩具” (toys) and “融合” (integration) are also relevant terms, pointing towards the use of play in therapeutic and educational contexts and the goal of integrating children with autism into various social environments. The word “希望” (hope) reveals the community’s positive outlook for their children’s future.

An illustrative example from the dataset is a post that states the following:

(6) “自闭症儿童最好的干预方法就是家长去学习，调整孩子的生理基础达到接近正常孩子的标准，解决好吃喝拉撒睡运动，在特殊教育心理学的指导下开展行为训练和融合.” (translated: “The best intervention method for children with autism is for parents to learn and adjust the child’s physiological foundation to standards close to that of typical children, manage eating, drinking, excreting, sleeping, and exercising well, and carry out behavioral training and integration under the guidance of special education psychology.”).

This post emphasizes the proactive role of parents in learning and conducting interventions, as well as the importance of establishing a strong physiological and psychological foundation for children with autism. Terms such as “干预” (intervention), “特殊教育” (special education), and “融合” (integration) highlight the tailored educational approaches to involve children with autism in educational settings that cater to their unique needs. The recurrent mention of “家长” (parents) and “儿童” (children) within this topic illustrates the focus on the family unit and its important role in the educational journey of a child with autism.

The analysis of Topic 2 highlights a community deeply involved in exploring and sharing knowledge about ASD rehabilitation and behavioral management in education settings. The discussion on recovery and training reflects an active pursuit of solutions and support among group members, which is crucial for individuals and families managing ASD.

### 3.3. Early Childhood Symptoms and Family Interaction

**Figure 4:**
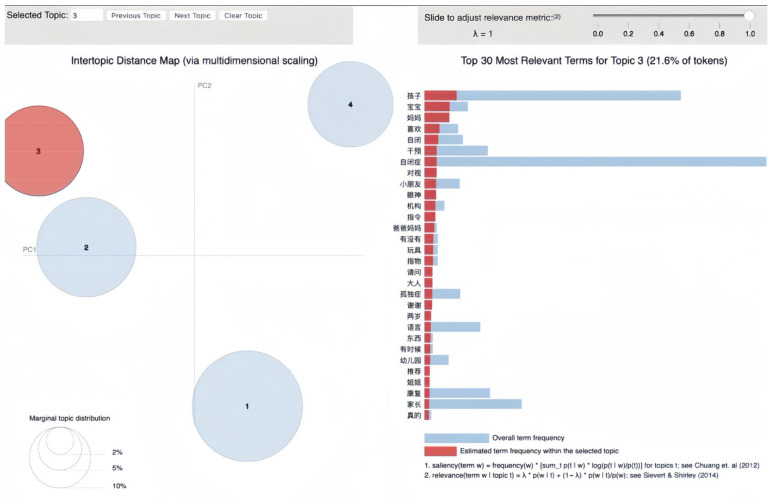
Visualization of Topic 3: early childhood symptoms and family interaction.

Topic 3 forms a distinct cluster in the corpus, representing 21.6% of the tokens, as shown on the intertopic distance map in Figure 4. The bar chart displays its top 30 most relevant terms, comparing their frequency within the topic to their overall distribution in the dataset.

The salient terms in Topic 3, such as “孩子” (children), “宝宝” (baby), “自闭”(autism), and “妈妈” (mother), underscore the focus on early recognition of ASD and the dynamics of family interaction. These terms reflect discussions within the community about detecting early signs of ASD, such as issues with “对视” (eye contact), “指物” (pointing), and “语言” (language development). The frequent mention of “干预” (intervention) and “康复” (rehabilitation) alongside “爸爸妈妈” (parents) highlights the role of parental involvement in early-intervention strategies and therapeutic processes. 

Furthermore, terms such as “喜欢” (enjoy), “玩具” (toys), and “情感” (emotion) reveal involving ASD children in activities that foster emotional bonding and communication within the family unit. The presence of “幼儿园” (kindergarten) shows discussions on involving children with ASD in school settings, emphasizing the importance of socialization from an early age.

The inclusion of keywords such as “自闭症” (ASD) and “孤独症” (autism, literally “loneliness disease”) also point to a broader examination of ASD itself, providing a backdrop against which family interactions and early childhood symptoms are explored. The community’s engagement with research and evidence-based practices is reflected in terms such as “机构” (institutions), “研究” (research), and “推荐” (recommend), suggesting an active pursuit of the latest findings in ASD therapy and care.

Additionally, personal and emotive terms such as “真的” (really) and “谢谢” (thank you) reflect the supportive nature in the online discussions, where families share experiences, seek advice, and express gratitude for the guidance received.

At the general level, the thematic feature of Topic 3 indicates that the ASD support groups are a crucial resource for families, offering a space to share personal experiences, gain insights from others’ journeys, and find comfort in a community that understands the unique challenges of raising a child with ASD. The findings from this topic serve as a testament to the power of community and the shared pursuit of improved quality of life for children with ASD and their families.

An example post from the forum that supports the theme of Topic 3 is shown below:

(7) “二岁半了男宝，语言还是没有进步，有主动语言，有需求会喊我，拉屎撒尿都会说，你我他不知道。吃饭自己可以，平时比较依赖我，我要是走开他的视线，他会找我，会大喊妈妈，会一直喊，直到我出来，真找不到他会哭，认知很好，蔬菜水果动物汽车类都知道，不要没有知道了都会表达，理解能力也很好，一步指令都能理解，就是成段的语言不会说，特别挑食，爱吃肉，不爱吃菜，动画片也爱看，里面的人物都会说，都认识，在家真不知道怎么教，因为疫情也不敢带他玩，感觉在家里越呆越傻。” (translated: “My two-and-a-half-year-old son has not made progress in language ability; he has some initiative language, for example, he calls me when he needs something and can indicate when he needs the toilet, but does not understand personal pronouns such as ‘you’, ‘I’, and ‘he’. He can eat by himself and generally relies on me a lot. If I step out of his sight, he will look for me and call out ‘mom’ continuously until I appear. If he really can’t find me, he will cry. His cognition is good; he knows vegetables, fruits, animals, and vehicles, and can express his needs. His understanding is also good; he can follow one-step instructions but doesn’t speak in sentences. He is particularly picky with food, likes meat but not vegetables, and enjoys cartoons, recognizing and naming the characters. I am unsure how to teach him at home, especially since we can’t go out to play due to the pandemic; it feels like staying at home is not helping his development but making him sillier.”).

This post highlights key issues of concern to parents of young children with ASD, including language delays, eating habits, and the need for constant parental presence and reassurance. It reveals the complexity of early ASD symptom recognition and the nuanced ways families respond to their child’s needs. The discussion is rich in descriptions of day-to-day interactions and the parental strategies in supporting children’s development. Such post underscores the emotional and practical challenges families face and highlights the importance of support and knowledge-sharing within the community.

### 3.4. Educational Resources and Community Support

**Figure 5:**
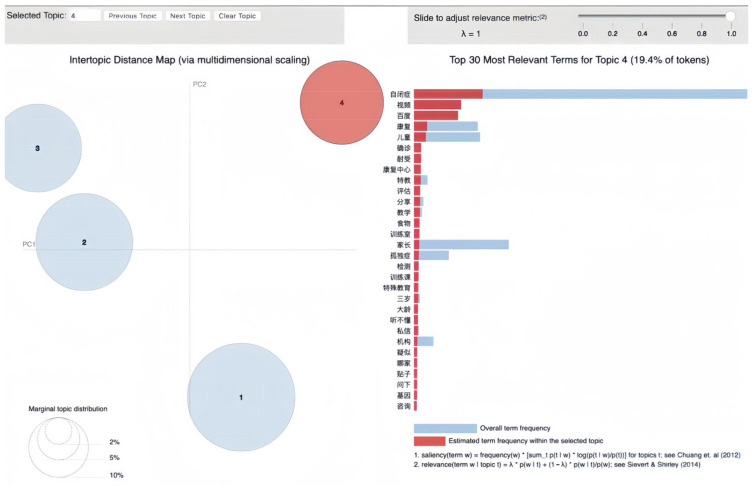
Visualization of Topic 4: educational resources and community support.

Topic 4 is a substantial and isolated topic on the intertopic distance map, a unique theme comprising 19.4% of tokens in the corpus. As shown in Figure 5, Topic 4 underscores the online support group’s engagement with evidence-based approaches to ASD, reflecting a proactive community seeking to understand and address the condition through various interventional and educational resources.

Looking at Figure 5, the bar chart of the top 30 most relevant terms for Topic 4, predominantly highlighted with red bars, demonstrates concerns about educational and support mechanisms within the autism community. The top keywords paint a picture of a community deeply invested in the exchange of information and resources that can empower families and individuals dealing with autism. Red bars in Figure 5 prominently feature “自闭症” (autism), “视频” (video), and “百度” (Baidu), suggesting that digital media and online platforms are effective ways for sharing and accessing autism-related educational content. The high frequency of “康复” (rehabilitation) and “康复中心” (rehabilitation center) denotes a strong community focus on resource centers and facilities, emphasizing the practical aspects of care and support.

The presence of terms such as “儿童” (children), “确诊” (diagnosed), and “评估” (assessment) indicates concerns surrounding the process of diagnosing and understanding children’s specific needs. “特教” (SE, special education) and “特殊教育” (special education) underscore the specialized teaching methods and educational resources tailored for children with ASD.

Furthermore, keywords such as “家长” (parents) and “孤独症” (autism, literally “loneliness disease”) reflect the crucial role of familial support and the individual experiences of those living with autism. “分享” (share), “帖子” (posts), and “私信” (private messages) indicate a rich exchange of personal stories, tips, and direct communication within the community, fostering a sense of solidarity and shared purpose.

The visualization also suggests that while “教学” (teaching) and “训练课” (training classes) are discussed, other elements such as “食物” (food) and “基因” (genes) indicate an interdisciplinary approach to understanding and managing ASD, including dietary considerations and genetic research.

Topic 4 delves into the realm of educational resources and community support, highlighting the community’s pursuit of information regarding educational institutions, therapy centers, and the overarching support structure available for children with ASD and their families. This topic encapsulates the collective concern over choosing the right educational path and support mechanisms for children diagnosed with ASD. Two examples exemplify the core issues and inquiries prevalent within this topic.

(8) “各位家长好，我家孩子刚被确诊为自闭症，打算找机构，请问怎么知道一个机构是否正规，里面的老师是不是好老师，有能力呢？谢谢大家.” (translated: “Hello fellow parents, my child has just been diagnosed with autism, and I am looking to find an institution. How can I know if an institution is legitimate, and if the teachers there are good and capable? Thank you all.”).

This post underscores the anxiety and desire for guidance that parents feel upon receiving an ASD diagnosis for their child, emphasizing the critical need for reliable information on educational and therapeutic institutions.

(9) “儿子还是不跟小朋友玩，在幼儿园能够安坐，但是显得不合群，到底去机构还是继续在幼儿园？每天都焦虑，真不知道该怎么办了.” (translated: “My son still does not play with other children. He can sit quietly in kindergarten but seems out of place; he cannot socialize. Should we go to a specialized institution or continue to stay in kindergarten? I am anxious every day and really don’t know what to do.”).

This example depicts parents’ dilemmas in deciding between mainstream educational environments and specialized institutions designed for children with ASD, reflecting the deep concerns over social integration and appropriate educational settings.

These examples, which mostly comprise parents’ concerns, portray the active search for educational strategies, support services, and community advice tailored to children with ASD. They represent a crucial component of the discourse within the ASD support groups, demonstrating the vital role of shared knowledge and experiences in aiding decision-making processes for families. The focus on educational resources and community support within Topic 4 signals a collective effort to navigate the challenges posed by ASD, emphasizing the importance of community engagement, peer support, and access to quality education and care for children with ASD.

## 4. Discussion

This study examined how discourse within online ASD communities enhances our understanding of social media’s role in providing support, facilitating information exchange, and shaping societal perceptions of ASD. Using LDA topic modeling on text data from Baidu Tieba, we identified four key themes in Chinese ASD support groups’ online discussions: intervention and therapy, early educational journey, early symptom detection and family interaction, and access to educational resources and community support. These themes indicate multi-dimensional concerns of Chinese ASD support groups, highlighting in particular their need of evidence-based knowledge regarding therapeutic approaches, early schooling life, symptom identification, and family intervention. Moreover, discussions on ASD within Baidu Tieba further underscore the importance of accessible educational resources and robust social support for autistic individuals and their families.

### 4.1. Topics Among ASD Support Groups

The discourse patterns reveal proactive, practical, and evidence-oriented discussions among members of ASD support groups. The emphasis on treatment and recovery in Topic 1 shows a proactive stance towards ASD, supporting the literature stressing the importance of early and sustained intervention and therapy in ASD management (W. Dunn et al., 2012; Kasari et al., 2012; Landa, 2007; Smith & Iadarola, 2015). The prominence of this topic within the ASD support group also mirrors previous studies highlighting the centrality of treatment-related information in caregiver discussions (W. Dunn, 2011; Zhao et al., 2019). Topic 2 focuses on early education for autistic children, which integrates schooling settings, the home environment, and healthcare systems. The discussions captured in this topic resonate with prior research that underscores developing inclusive learning environments for young autistic children so that they can receive appropriate teaching in speaking, communication, behavioral and social skills (Guldberg, 2010). In Topic 3, the online communication about early symptom recognition and parental involvement in autism child development demonstrates the support group’s engagement with symptom management, scientific intervention, emotional support, and information seeking. This involvement is crucial as it empowers families with knowledge to make informed decisions about ASD care (Lorence, 2007), thereby echoing the calls for evidence-based treatment advocacy in the ASD community (Bellon-Harn et al., 2022; Reichow & Volkmar, 2010). The focus on educational resources and social support in Topic 4 indicates the importance of educational planning in raising children with autism (Simpson, 2005). China faces a significant gap in specialized educational programs and support services for children with autism. Mainstream schools lack the necessary educational resources and trained professionals to support autistic students (X. Li et al., 2024). Thus, the sharing of educational institutions, treatment centers, and the related healthcare services within the autism support community is essential for educational strategies and intervention among families with autistic children.

The topic-modeling-based discourse analysis demonstrates that ASD support groups on social media are not mere forums for emotional support but are pivotal in information exchange and collective learning. The active sharing of knowledge and experiences within the online groups can be seen as a form of collective coping mechanism, which contributes to bolstering community resilience and empowerment (Kientz & Dunn, 2012). The convergence of the four topics indicates a multifaceted, community-driven approach to ASD, combining personal stories, practical advice, and evidence-based knowledge. This directly aligns with Coulson’s (2005) concept of “informational support” in online health communities and mirrors the core discursive functions—practical advice, emotional support, and personal narratives—identified in discourse analyses of similar Western social media platforms such as Facebook (Zhao et al., 2019). The information support among the ASD community, in particular sharing of knowledge and experiences among parents, can challenge stigmatizing beliefs and misconceptions toward ASD (Lewis, 2023; X. Li et al., 2024).

The online communication and interaction within ASD support groups, as shown in topics such as intervention strategies and educational resources, has the potential to extend beyond personal support to influence broader social awareness, healthcare practices, and policymaking concerning ASD (Larnyo et al., 2024). Hence, the proactive information sharing and knowledge dissemination within these groups can lead to an increased societal understanding of ASD. By echoing the community’s voice, online platforms such as Baidu Tieba can shape public opinions and contribute to the destigmatization of ASD (Gillespie-Lynch et al., 2015; Zhao et al., 2019; Q. Zhou et al., 2023). The prevalence of evidence-based discussions may also serve as a persuasive tool for advocating changes in healthcare practices, emphasizing the necessity for early-intervention programs and accessible therapeutic services (W. Dunn, 2011; Walsh et al., 2018). Furthermore, the detailed social media discourse concerning educational strategies and family experiences within ASD support groups can inform policymakers of the community’s specific needs. By providing a window into the daily lives of individuals with ASD and their families, the online communication among ASD support groups can facilitate the development of tailored educational and healthcare policies for the ASD population (Davidson & Orsini, 2013; Larnyo et al., 2024). At the general level, the multi-thematic social media discourse positions ASD online groups as integral nodes in the information journeys of caregivers and advocates (Duffin et al., 2025), although the discussions can sometimes foster in-group tensions along with support (Skafle et al., 2024). Ultimately, these online forums offer a powerful, collective knowledge base capable of informing individual decisions and driving systemic change concerning ASD-related practices and policies.

In light of comparison, the online discussion on ASD within the Chinese context exhibits both similarities and differences with global patterns reported in the literature. The strong orientation towards evidence-based knowledge and treatment discussions in the present study resonates with findings from Western contexts that highlight scientific information sharing within autism support communities (Bellon-Harn et al., 2022; Larnyo et al., 2024; Nguyen et al., 2014). However, the stress on educational interventions in our study reflects region-specific priorities, such as accessing and optimizing within local educational and healthcare frameworks. This emphasis stands in contrast to the greater focus on adult diagnosis and neurodiversity identity observed in discussions within English-language forums (e.g., Duffin et al., 2025; Larnyo et al., 2024). The comparative perspective indicates that while the fundamental need for informational support is universal, its specific manifestations are shaped by local sociocultural context.

It is worth mentioning that our findings predominantly provide insight into parent and caregiver views and opinions, rather than direct evidence on autism interventions or lived experience. In the present study, the discussions reveal what parents believe to be important, such as early socialization and establishing a strong physiological and psychological foundation for their children. This indicates a strongly held belief within this caregiver community but may not itself constitute evidence for the efficacy of these therapeutic approaches. Hence, our study captures the discourse of belief and prioritization among parents, not objective outcomes. This caregiver-dominated perspective is a typical feature of our data, as autistic individuals themselves are somehow absent from these discussions. This absence mirrors a broader tension noted in the literature, where online platforms crucial for caregiver support may marginalize voices of autistic individuals (Skafle et al., 2024), and thus may lead to a representation gap in ASD discourse. While parents seek and share peer-led knowledge (Duffin et al., 2025; Lorence, 2007), the lack of direct autistic voices means that the discourse may not fully reflect the priorities, preferences, or experiences of autistic individuals—a critical consideration for researchers, clinicians, and policymakers who might engage with social media. Future research should integrate these caregiver insights with communication patterns of autistic individuals to achieve a comprehensive understanding of the needs of the ASD community (e.g., Duffin et al., 2025).

Overall, the discourse within ASD support groups embodies a powerful collective knowledge base that can inform and influence not only individual decisions but also social attitudes, healthcare practices, and policymaking. As these online communities continue to grow and participate in multifaceted communication, their collective voice could become a force in advocating for the rights and needs of individuals with ASD, ultimately contributing to a more inclusive and supportive society (Coulson, 2005; Kientz & Dunn, 2012; Q. Zhou et al., 2023).

### 4.2. Sociocultural Factors in Chinese Social Media and Implications

The collectivist nature of Chinese society, which emphasizes group harmony and social stability, may influence how individuals express themselves in online forums (Fan & Ko, 2025; Hofstede, 2001). The cultural emphasis on normalcy and conformity in China makes parents of autistic children bear more pressure than parents from Western cultures, which often leads to the misunderstanding of autistic individuals (X. Li et al., 2024). Chinese parents are very likely to be impacted by the concept of “saving face” and group identification, and thus may adopt conservative or covert strategies in dealing with their children’s disability (Fan & Ko, 2025). As a result, the perception of ASD and disability in China has its peculiarities, marked by stigma and lack of awareness (Tang & Bie, 2016; Q. Zhou et al., 2023), which may lead families to hesitate in openly sharing their struggles due to fear of social judgment or isolation. The rapid development of digital technology in China has led to a rise of health information and services on social media platforms (X. Li et al., 2024; Q. Zhou et al., 2023), potentially contributing to the high volume of online discussions observed in ASD support groups, as the availability of these resources may empower users to focus on sharing practical advice and information. In addition, government policies and initiatives surrounding healthcare and disability, such as the push for more inclusive education and better healthcare services for individuals with ASD (Fan & Ko, 2025; X. Li et al., 2024; Zhang et al., 2024), could also influence the constructive nature of the conversations observed in these ASD support groups.

These factors together form a unique sociocultural ecosystem within which the Chinese ASD support groups share their ideas. Understanding these factors is vital for interpreting the topics expressed in these groups and for designing interventions and support mechanisms that are culturally and contextually appropriate.

Based on the sociocultural factors identified within the Chinese social media context, several implications are proposed. With respect to collectivism, fostering an environment that emphasizes empathetic understanding and collective support in online forums could encourage more open exchanges of personal experiences and challenges. Implementing moderated discussions that prioritize respect and confidentiality may mitigate concerns related to stigma and social judgment, thereby promoting a more inclusive dialog around ASD (Tang & Bie, 2016). In addition, creating anonymous posting options could encourage users to share their struggles without fear of social exclusion. Furthermore, social media platforms can collaborate with healthcare professionals to provide verified information and support, which can maximize the benefits of online ASD communities (Sun et al., 2013; Q. Zhou et al., 2023). Additionally, aligning with government initiatives, social media forums could host discussions and workshops that educate and inform about recent policies, rights, and services available to individuals with ASD and their families, fostering a more informed and engaged community (Fan & Ko, 2025; Zhang et al., 2024). These implications can not only strengthen communication within ASD support groups but also contribute to the broader social inclusion of individuals with ASD and their families.

### 4.3. Limitations and Future Research

This study has several limitations. First, the exclusive reliance on data from Baidu Tieba, a single platform, restricts the generalizability of our findings. Communication practices and community views may vary across different social media platforms and offline contexts (Boyd & Ellison, 2007). Second, the social media data primarily shows the perspectives of caregivers, especially parents, which may not fully represent the broader ASD-affected population in China. Thus, the findings are skewed towards users comfortable with technology and public sharing, potentially overlooking those who do not participate in online forums. A critical shortcoming is the underrepresentation of voices from individuals with ASD themselves. Due to factors such as social camouflage, autistic individuals may be less visible in these online spaces, leading to a lack of their direct lived-experience stories (Hull et al., 2017). Third, while data included user locations and timestamps, this study did not utilize this information for spatiotemporal analysis of posting patterns (i.e., when most of the posts about specific topics were spoken about and where most of them were sent from). Furthermore, the data-cleaning process, though necessary, may have removed part of the textual context, potentially influencing the depth of thematic interpretation.

In light of these challenges, future research can expand datasets to multiple online platforms in order to present a multi-dimensional view of the ASD community. Furthermore, analyses of social media data can be combined with methods such as interviews and observational studies. This convergent approach can capture the firsthand lived experiences and perspectives of autistic individuals. Additionally, the spatiotemporal metadata available in social media datasets should be considered in future analyses to reveal discursive patterns concerning geographical factors. Longitudinal designs are also needed to show how discussions within the ASD community shape shifts in policy, healthcare, and public awareness. Finally, comparative cross-cultural studies can be conducted to build a more comprehensive understanding of communication within ASD support groups, integrating perspectives from both Chinese sociocultural contexts and global frameworks.

## 5. Conclusions

Based on LDA topic modeling of the ASD discourse on Baidu Tieba, this study identified four topics among Chinese ASD support groups: autism intervention and therapy, early childhood autism and education, early symptoms and family interaction, and educational resources and community support. These topics demonstrate that social media platforms such as Baidu Tieba serve as essential ASD communities not only for information sharing and emotional support but also as online spaces of collective advocacy. The discussions concerning scientific knowledge on ASD and personal experiences empower parents and caregivers with the knowledge and communal solidarity to spell out their needs more effectively. This empowerment, in turn, enables them to make informed demands on the healthcare and educational systems, and thereby can guide policymaking in improving the well-being of people with autism and their caregivers. By amplifying a collective voice, online forums raise the visibility of systemic gaps, such as the need for more accessible early-intervention services or special educational resources. Consequently, individual struggles can be transformed into public efforts that can push policymakers to implement more responsive and family-centered support frameworks.

The insights in this study underscore the multifunctional role of online platforms in enhancing community resilience and informing public policy, while also highlighting certain limitations, such as the predominance of caregiver perspectives and the underrepresentation of autistic voices. Thus, the findings offer practical implications for the development of targeted interventions and support systems tailored to individuals with ASD and their families, both within and beyond the digital realm.

## Figures and Tables

**Table 1:** Naming of themes under each topic based on LDA topic modeling.

	Topic 1	Topic 2	Topic 3	Topic 4
Themes	Autism Intervention and Therapy	Early Childhood Autism and Education	Early Childhood Symptoms and Family Interaction	Educational Resources and Community Support
Keywords	儿童 (children), 语言 (language), 康复 (rehabilitation), 家长 (parents), 干预 (intervention), 训练 (training), 治疗 (treatment), 患儿 (pediatric patients), 星康 (Xingkang “Star Health”), 康复训练 (rehabilitation training), 核医学 (nuclear medicine), etc.	老师 (teacher), 小朋友 (kids), 喜欢 (like), 幼儿园 (kindergarten), 宝宝 (baby), 医生 (doctor), 儿子 (son), 交流 (communicate), 干预 (intervention), 刻板 (stereotypical), 说话 (speak), 特教 (special education), 爸爸 (dad), 感觉 (sense), 机构 (institute), 统合 (integration)	妈妈 (mother), 喜欢 (enjoy), 干预 (intervention), 对视 (eye contact), 小朋友 (kids), 眼神 (eye contact), 机构 (institution), 指令 (instruction), 爸爸妈妈 (parents), 玩具 (toy), 指物 (point at things), 大人 (adults)	视频 (video), 百度 (Baidu), 康复 (rehabilitation), 确诊 (diagnosed), 耐受 (tolerate), 康复中心rehabilitation center), 特教 (special education), 评估 (evaluation), 分享 (share), 教学 (teaching), 食物 (food), 训练室 (training room), 私信 (private message), 机构 (institution), 基因 (gene), 咨询 (consult)

## Data Availability

The social media data in this study can be accessed from the following link: https://doi.org/10.57760/sciencedb.30428.
